# First detection of dengue and chikungunya viruses in natural populations of *Aedes aegypti* in Martinique during the 2013 – 2015 concomitant outbreak

**DOI:** 10.26633/RPSP.2017.63

**Published:** 2017-07-06

**Authors:** Laurence Farraudière, Fabrice Sonor, Said Crico, Manuel Étienne, Laurence Mousson, Rodolphe Hamel, Dorothée Missé, Anna-Bella Failloux, Frédéric Simard, André Yébakima

**Affiliations:** 1 Centre de Démoustication et de Recherches Entomologiques/Lutte antivectorielle Collectivité Territoriale et Agence Régionale de Santé Fort-de-FranceMartinique France Centre de Démoustication et de Recherches Entomologiques/Lutte antivectorielle, Collectivité Territoriale et Agence Régionale de Santé, Fort-de-France, Martinique, France.; 2 Arboviruses et Insect Vectors Department of Virology, Pasteur Institute Paris France Arboviruses et Insect Vectors, Department of Virology, Pasteur Institute, Paris, France.; 3 Maladies Infectieuses et Vecteurs: Écologie Génétique, Évolution et Contrôle, Université de Montpellier Montpellier France Maladies Infectieuses et Vecteurs: Écologie, Génétique, Évolution et Contrôle, Université de Montpellier, Montpellier, France.

**Keywords:** Aedes, culicidae, dengue, chikungunya virus, vector control, Martinique, French West Indies, Caribbean Region, Aedes, culicidae, dengue, Virus chikungunya, control de vectores, Martinica, Indias Occidentales, Región del Caribe

## Abstract

Dengue and chikungunya viruses are transmitted by Aedes mosquitoes. In Martinique, an island of the French West Indies, Aedes aegypti is the suspected vector of both arboviruses; there is no Aedes albopictus on the island. During the concomitant outbreak of 2013 – 2015, the authors collected wild A. aegypti populations, and for the first time, detected dengue and chikungunya viruses in field-collected females. This paper demonstrates the mosquito’s role in transmission of both dengue and chikungunya on the island, and also highlights a tool that public health authorities can use for preventing outbreaks.

## RESUMEN

Los virus del dengue y del chikungunya se transmiten a través de los mosquitos del género *Aedes.* Se da por supuesto que en Martinica, en las Antillas francesas, *Aedes aegypti* es el vector de ambos arbovirus, puesto que en la isla no hay *Aedes albopictus.* Durante el brote concomitante del 2013 al 2015, los autores recogieron muestras de poblaciones salvajes de *A. aegypti* y, por primera vez, detectaron virus del dengue y del chikungunya en las hembras obtenidas en el terreno. En el presente artículo se demuestra que el mosquito actúa en la isla como transmisor tanto del dengue como del chikungunya y se describe, además, una herramienta que las autoridades de salud pública pueden utilizar para prevenir los brotes.

Martinique is a French overseas department located in the Lesser Antilles of the French West Indies with a population of 400 000. On this island, six major dengue fever (DF) outbreaks have occurred in the past 20 years (1). In December 2013, while a DF outbreak was ongoing, circulation of chikungunya virus (CHIKV) was also reported. This first-ever reported outbreak of CHIKV in Martinique lasted 14 months and recorded 72 520 clinically-suspected cases and 83 deaths (2). Reporting of clinical cases and assessment of mosquito abundance are indicative of the potential role of *Aedes aegypti* as the main vector. The invasive Asian tiger mosquito, *Aedes albopictus,* has not been reported from the island to date; however, evidence for natural virus infection in local *A. aegypti* populations is lacking. This study fills the gap by reporting on detection of dengue virus (DENV) and CHIKV in field-collected *A.aegypti* females, and corroborates theimplication of *A. aegypti* in the DENV/CHIKV outbreak on Martinique in 2013–2015.

## MATERIALS AND METHODS

From September 2013–July 2014, mosquitoes were collected throughout Martinique from the 162 houses where confirmed and/or suspected DENV and/or CHIKV cases had been reported. After confirmation of cases, entomological investigations were initiated by mosquito-control field teams. Adult mosquitoes were collected with a battery-powered backpack aspirator (Centers for Disease Control and Prevention, Atlanta Georgia, United States), both indoors and outdoors at the houses of reported cases, as well as from neighboring homes (3). Collected mosquitoes were brought to the laboratory, killed at -20°C, and separated by sex and locality. Abdomens of females were removed with a needle to avoid contamination with human blood. The mosquitoes were then stored in pools of 10 specimens at -80°C until testing.

To investigate whether viruses can be transmitted vertically in the field (i.e., from an infected female to its progeny), pupae were also collected from all productive mosquito breeding places detected in- and outdoors, in the vicinity of human reported cases. Pupae were reared to adults in insectaries. These specimens were sorted by sex and locality, and stored in pools of 10 mosquitoes at -80°C. Mosquito pools were homogenized in a solution (500 µL) constituted of Dulbecos Modified Eagle’s Medium (DMEM 1x) with Glutamax (ThermoFisher Scientific, Courtaboeuf, France) and Pen/Strep (1%) using a mixer (Retsch, MM301). After centrifugation (1 000 stroke for 2 minutes at 4°C), 150 µL of supernatant was filtered on a QIAshredder column (Qiagen, Courtaboeuf, France) to clear samples. Pools were transferred into vials containing 600 µL of lysis solution and extraction of viral ribonucleic acid (RNA) was performed using QIAamp Viral RNA Mini kit (Qiagen, Courtaboeuf, France) according to manufacturer’s recommended procedures. Reverse transcription was performed with 14 µL of RNA template using Moloney murine leukemia virus (M-MLV) reverse transcriptase (Promega, Charbonniers, France) according to the manufacturer’s instructions. Quantitative Real-Time Polymerase Chain Reaction (qRT-PCR) was then performed using 2 µL of complementary DeoxyriboNucleic Acid (cDNA) with specific primers targeting glycoprotein E1 for CHIKV and 3’NC region (pan-system) for DENV (4) and 10 µL of Maxima probe/ROX qPCR master mix (Fermentas, Saint Remy les Chevreuses, France) in a final reaction volume of 20 µL. The cycling conditions were 45 cycles at 95°C for 15 seconds, 60°C for 20 seconds, and 72°C for 30 seconds.

## RESULTS

A total of 414 pools of field-collected mosquitoes was obtained, including 313 pools of captured adults (146 pools of males and 167 pools of females) and 101 pools of emerged adults (49 pools of females and 52 pools of males). All *A. aegypti* specimens were included in the study and all pools were tested for CHIKV and DENV using qRT-PCR. The presence of CHIKV was detected in 11 pools of females captured as adults in the field. No pool of males or emerged adults (males and females) was positive for CHIKV. Of the 414 pools, 5 were positive for DENV. Of these positive pools, 4 were from females captured as adults in the field, and 1 was from a pool of emerged females. No pool of male mosquitoes (captured or emerged) was positive for DENV, nor were any positive for both viruses.

## DISCUSSION

This study reports on the first time that DENV and CHIKV infections were found in natural populations of *A. aegypti* on Martinique. This finding was expected, since no other putative DENV and CHIKV vector is present on the Island. Vector competence of *A. aegypti* for DENV and CHIKV has indeed been proven in the laboratory, including among populations from Martinique (5, 6). Furthermore, the species was incriminated as a major vector of both viruses in previous epidemics in the area, including in Cuba and Puerto Rico, as well as in continental Central and South America, including Brazil and Mexico (7). These study results, therefore, are consistent with previous evidence pointing to the major role that *A. aegypti* plays as a DENV and CHIKV vector in the Caribbean, and provides evidence for its involvement in transmitting both viruses in Martinique during the 2013–2015 epidemic.

It is now important that, both viruses be isolated from positive mosquito pools, and sequenced. Because *A. albopictus* is not found on the Island, it is unlikely that other mosquito species would have contributed significantly to transmission. However, future studies should include virus detection in a more representative sample of the *Culicidae* fauna of Martinique island, to ascertain if *A. aegypti* is the Island’s only DENV and CHIKV vector of epidemiological importance.

Vertical transmission of DENV by *A. aegypti* has been demonstrated in the laboratory (8). Our findings indicate that it occurred in the field, as well during the 2013–2015 epidemic in Martinique. Vertical transmission may contribute to the dynamics of an epidemic burst, as well as to the maintenance of endemic DENV on the Island during inter-epidemic periods. It may also be a cause of cyclic re-emergence of dengue outbreaks on the Island.

Future studies are needed to further explore this hypothesis and assess whether the virus that is transmitted vertically is infective and can be further transmitted to human hosts. On the other hand, the results of the present study did not provide evidence for vertical transmission of CHIKV, although this phenomenon has been observed in natural populations of *A. aegypti* in Mexico (9).

Recent experiments have shown *A. aegypti* from Martinique to be a competent vector for Zika virus (ZIKV) under controlled laboratory conditions (10). Because ZIKV is rapidly spreading throughout the continental Americas and the Caribbean, enforced surveillance and control of *A. aegypti* should be implemented throughout Martinique to prevent local epidemics. When routinely implemented, arbovirus detection could indeed provide a powerful health monitoring indicator, helping public health authorities and mosquito-control staff predict, prevent, and control diseases outbreaks.

In conclusion, *A. aegypti* represents a major threat to public health in Martinique. Innovative strategies and tools for monitoring and controlling this mosquito are needed to mitigate the risk of future outbreaks.

## Acknowledgements

We are grateful to the field operators of the Centre de Démoustication et de Recherches Entomologiques/Lutte antivectorielle, (Collectivité Territoriale et ARS Martinique), for their help and useful suggestions during the outbreak investigations. This work was funded by the Conseil Régional and Conseil Général de la Martinique.

## Disclaimer.

Authors hold sole responsibility for the views expressed in the manuscript, which may not necessarily reflect the opinion or policy of the *RPSP/PAJPH* and/or PAHO.
